# Supplementary material for: Modeling glioblastoma heterogeneity as a dynamic network of cell states
Source: Mol Syst Biol. 2021 Sep 16;17(9):e10105. doi: 10.15252/msb.202010105 (PMC8444284; doi:10.15252/msb.202010105)
Supplement: Supplementary file 6 — Source Data for Figure 5 [file MSB-17-e10105-s004.zip › Figure5A_sourcedata/GSEA_3017/hallmarks_stateF.GseaPreranked.1621934628066/HALLMARK_MITOTIC_SPINDLE.html]

Details for gene set HALLMARK\_MITOTIC\_SPINDLE[GSEA]

|  || Dataset | state33017 |
| Phenotype | NoPhenotypeAvailable |
| Upregulated in class | na\_neg |
| GeneSet | HALLMARK\_MITOTIC\_SPINDLE |
| Enrichment Score (ES) | -0.68925685 |
| Normalized Enrichment Score (NES) | -4.049906 |
| Nominal p-value | 0.0 |
| FDR q-value | 0.0 |
| FWER p-Value | 0.0 |
Table: GSEA Results Summary

  

Fig 1: Enrichment plot: HALLMARK\_MITOTIC\_SPINDLE      
 Profile of the Running ES Score & Positions of GeneSet Members on the Rank Ordered List

  

| PROBE | GENE SYMBOL | GENE\_TITLE | RANK IN GENE LIST | RANK METRIC SCORE | RUNNING ES | CORE ENRICHMENT || 1 | APC |  |  | 169 | 0.632 | -0.0631 | No |
| 2 | PDLIM5 |  |  | 223 | 0.572 | -0.0745 | No |
| 3 | SPTBN1 |  |  | 349 | 0.487 | -0.1208 | No |
| 4 | STK38L |  |  | 482 | 0.440 | -0.1713 | No |
| 5 | FGD4 |  |  | 503 | 0.434 | -0.1707 | No |
| 6 | ARHGAP29 |  |  | 627 | 0.390 | -0.2182 | No |
| 7 | DOCK4 |  |  | 668 | 0.380 | -0.2280 | No |
| 8 | SPTAN1 |  |  | 815 | 0.344 | -0.2870 | No |
| 9 | NEDD9 |  |  | 817 | 0.344 | -0.2798 | No |
| 10 | DST |  |  | 896 | 0.327 | -0.3081 | No |
| 11 | DLG1 |  |  | 1013 | 0.307 | -0.3543 | No |
| 12 | PCGF5 |  |  | 1045 | 0.301 | -0.3617 | No |
| 13 | KIFAP3 |  |  | 1064 | 0.298 | -0.3633 | No |
| 14 | KIF1B |  |  | 1276 | 0.271 | -0.4537 | No |
| 15 | ARHGEF7 |  |  | 1299 | 0.268 | -0.4578 | No |
| 16 | ARHGEF12 |  |  | 1379 | 0.259 | -0.4881 | No |
| 17 | OPHN1 |  |  | 1405 | 0.256 | -0.4938 | No |
| 18 | NOTCH2 |  |  | 1420 | 0.255 | -0.4945 | No |
| 19 | DYNLL2 |  |  | 1534 | -0.267 | -0.5402 | No |
| 20 | TUBGCP2 |  |  | 1566 | -0.277 | -0.5481 | No |
| 21 | CNTRL |  |  | 1610 | -0.288 | -0.5614 | No |
| 22 | TRIO |  |  | 1661 | -0.304 | -0.5774 | No |
| 23 | CEP57 |  |  | 1676 | -0.309 | -0.5769 | No |
| 24 | ARHGDIA |  |  | 1680 | -0.311 | -0.5713 | No |
| 25 | FARP1 |  |  | 1706 | -0.321 | -0.5755 | No |
| 26 | SASS6 |  |  | 1719 | -0.325 | -0.5737 | No |
| 27 | NEK2 |  |  | 1815 | -0.371 | -0.6089 | No |
| 28 | PCNT |  |  | 1854 | -0.392 | -0.6175 | No |
| 29 | CDK5RAP2 |  |  | 2012 | -0.503 | -0.6780 | Yes |
| 30 | SMC1A |  |  | 2021 | -0.513 | -0.6701 | Yes |
| 31 | NIN |  |  | 2032 | -0.523 | -0.6630 | Yes |
| 32 | TTK |  |  | 2047 | -0.533 | -0.6575 | Yes |
| 33 | KIF22 |  |  | 2053 | -0.541 | -0.6476 | Yes |
| 34 | CENPJ |  |  | 2054 | -0.544 | -0.6354 | Yes |
| 35 | INCENP |  |  | 2061 | -0.556 | -0.6257 | Yes |
| 36 | EPB41L2 |  |  | 2067 | -0.576 | -0.6151 | Yes |
| 37 | RACGAP1 |  |  | 2072 | -0.579 | -0.6040 | Yes |
| 38 | CKAP5 |  |  | 2086 | -0.623 | -0.5960 | Yes |
| 39 | PLK1 |  |  | 2087 | -0.624 | -0.5820 | Yes |
| 40 | PIF1 |  |  | 2088 | -0.627 | -0.5679 | Yes |
| 41 | BRCA2 |  |  | 2100 | -0.650 | -0.5584 | Yes |
| 42 | AURKA |  |  | 2110 | -0.673 | -0.5475 | Yes |
| 43 | KIF2C |  |  | 2136 | -0.756 | -0.5420 | Yes |
| 44 | KIF5B |  |  | 2137 | -0.756 | -0.5250 | Yes |
| 45 | NDC80 |  |  | 2142 | -0.775 | -0.5095 | Yes |
| 46 | FBXO5 |  |  | 2144 | -0.779 | -0.4925 | Yes |
| 47 | KIF23 |  |  | 2145 | -0.782 | -0.4750 | Yes |
| 48 | ANLN |  |  | 2149 | -0.797 | -0.4585 | Yes |
| 49 | SMC3 |  |  | 2150 | -0.800 | -0.4406 | Yes |
| 50 | ECT2 |  |  | 2163 | -0.846 | -0.4272 | Yes |
| 51 | KIF20B |  |  | 2165 | -0.852 | -0.4085 | Yes |
| 52 | BUB1 |  |  | 2170 | -0.860 | -0.3911 | Yes |
| 53 | KIF15 |  |  | 2185 | -0.915 | -0.3770 | Yes |
| 54 | KIF4A |  |  | 2186 | -0.921 | -0.3564 | Yes |
| 55 | LMNB1 |  |  | 2194 | -0.950 | -0.3383 | Yes |
| 56 | CCNB2 |  |  | 2200 | -0.983 | -0.3186 | Yes |
| 57 | KIF11 |  |  | 2206 | -1.008 | -0.2983 | Yes |
| 58 | CENPE |  |  | 2211 | -1.036 | -0.2769 | Yes |
| 59 | CDK1 |  |  | 2225 | -1.153 | -0.2570 | Yes |
| 60 | DLGAP5 |  |  | 2235 | -1.273 | -0.2326 | Yes |
| 61 | SMC4 |  |  | 2236 | -1.281 | -0.2039 | Yes |
| 62 | PRC1 |  |  | 2240 | -1.397 | -0.1740 | Yes |
| 63 | BIRC5 |  |  | 2242 | -1.469 | -0.1415 | Yes |
| 64 | TOP2A |  |  | 2246 | -1.562 | -0.1079 | Yes |
| 65 | NUSAP1 |  |  | 2247 | -1.600 | -0.0721 | Yes |
| 66 | TPX2 |  |  | 2248 | -1.609 | -0.0360 | Yes |
| 67 | CENPF |  |  | 2249 | -1.691 | 0.0018 | Yes |
Table: GSEA details [plain text format]

  

Fig 2: HALLMARK\_MITOTIC\_SPINDLE: Random ES distribution      
 Gene set null distribution of ES for **HALLMARK\_MITOTIC\_SPINDLE**
